# Not only for melanoma. Subcutaneous pseudoprogression in lung squamous-cell carcinoma treated with nivolumab: A case report

**DOI:** 10.1097/MD.0000000000005951

**Published:** 2017-01-27

**Authors:** Michal Sarfaty, Assaf Moore, Elizabeth Dudnik, Nir Peled

**Affiliations:** a Institute of Oncology, Davidoff Cancer Center, Rabin Medical Center, Petach Tikva; b Sackler Faculty of Medicine, Tel Aviv University, Tel Aviv, Israel.

**Keywords:** lung cancer, nivolumab, pseudoprogression, squamous-cell carcinoma

## Abstract

**Rationale:**

Pseudoprogression, that is, initial tumor growth followed by subsequent tumor regression, has been well described for immunomodulation therapy in melanoma patients. This phenomenon is not well defined in lung cancer. Nivolumab, an anti-PD-1 monoclonal antibody, was recently approved for nonsmall cell lung cancer (NSCLC) as a second-line therapy.

**Patient concerns and diagnosis:**

We present a patient with squamous NSCLC, suffering from multiple bone and subcutaneous metastases.

**Interventions:**

The patient was treated with nivolumab.

**Outcomes:**

A subcutaneous lesion in her upper back grew substantially after the first cycle of nivolumab, and later regressed, with marked improvement in all cancer sites.

**Lessons:**

Such pseudoprogression may serve to predict subsequent clinical response.

## 1 Introduction

Nivolumab, an antiprogrammed death 1 (PD-1) monoclonal antibody, was recently approved as second-line therapy for nonsmall cell lung cancer (NSCLC).^[1]^

PD-1 related therapies are an emerging field in cancer and currently, there are 3 approved therapies from this family (PD-1 blockade; nivolumab, pembrolizumab; programmed death ligand 1 [PDL-1] blockade; atezolizumab). PDL-1 is a ligand released by the tumor which inhibits the lymphocytes in the microenvironment. By blocking the PD-1 receptor (on the lymphocyte) or by scavenging the PDL-1, the immune system is enhanced and lymphocytes may interfere with the tumor tissue development. This process may cause local inflammation and physical growth. Once the immune system overcomes the tumor, fast shrinkage normally follows.^[2]^ This pseudoprogression may lead to misinterpretation of the patient's status and wrong clinical decisions.

We present here a case of pseudoprogression followed by dramatic regression of a subcutaneous metastatic lesion in a patient with squamous NSCLC treated with nivolumab. Pseudoprogression has been reported in 6.7% to 12% of patients with malignant melanoma treated by immunotherapy,^[3]^ while the manifestation of this phenomenon in lung cancer is still uncertain.

Ethics approval was not necessary in this case report.

## 2 Case presentation

A 68-year-old female smoker with a history of pulmonary embolism and diabetes mellitus was diagnosed with Stage IIIB (T4N2M0) squamous NSCLC. She was treated by definitive chemoradiotherapy with cisplatin and vinorelbine until September 2014. In October 2014, positron emission tomography–computed tomography (PET-CT) scan demonstrated a good response in the primary lesion; however, new metastases in the right adrenal gland and right femur developed and were irradiated in November 2014. A month later the disease progressed with development of multiple bone and subcutaneous metastases. At that point, the patient suffered from severe dyspnea and was oxygen-dependent. She received one cycle of carboplatin and gemcitabine followed by severe pancytopenia, and treatment was switched to nivolumab 3 mg/kg q14 days in January 2015.

**Figure 1:**
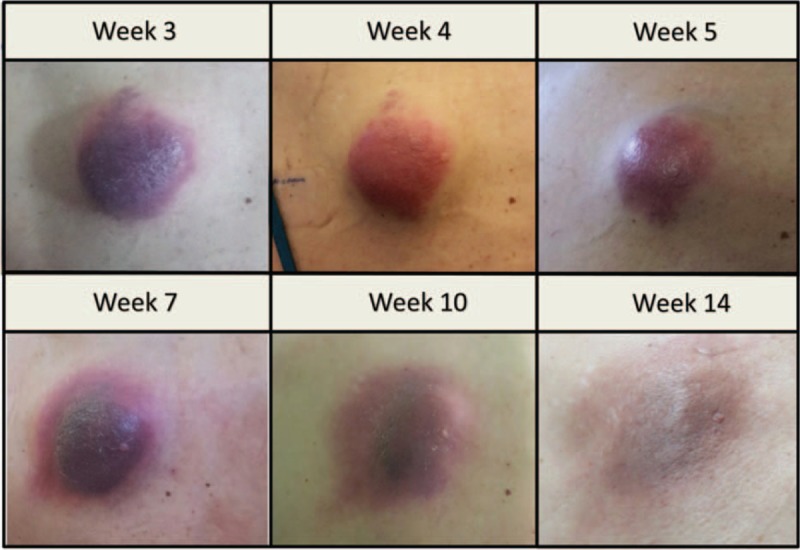
Response of a subcutaneous metastatic lesion to nivolumab, by week of treatment.

**Figure 2:**
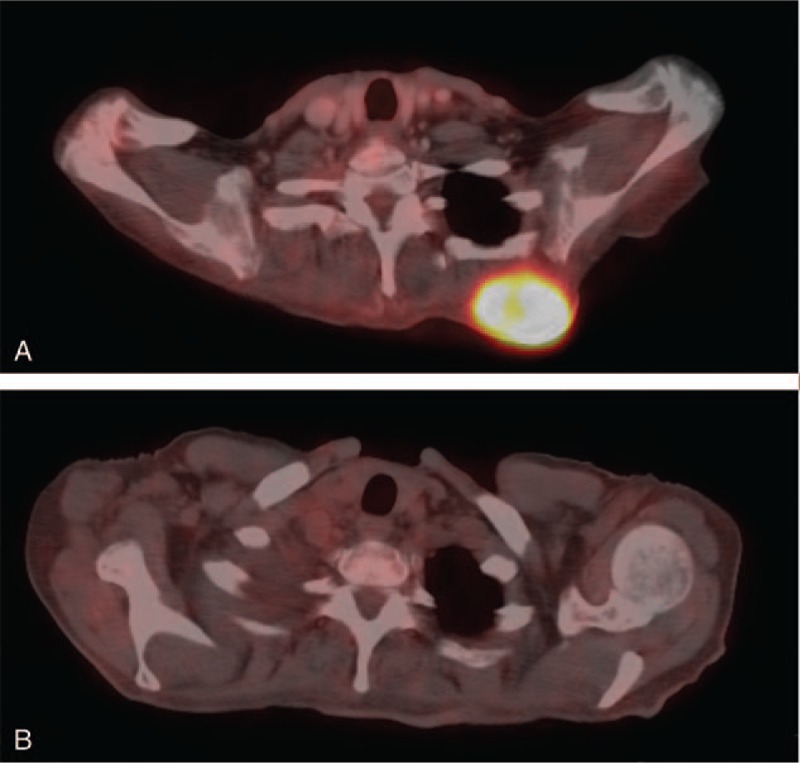
(A) PET-CT demonstrating a subcutaneous metastatic lesion, 2 months after starting nivolumab treatment (03/2015). (B) PET-CT demonstrating complete resolution of the lesion 5 months after starting nivolumab treatment (06/2015).

One week after the first cycle of nivolumab, a subcutaneous lesion in her upper back grew substantially, accompanied by severe pain and significant inflammatory reaction (Fig. 1). Other subcutaneous metastases grew slightly as well. After the second cycle of treatment marked symptomatic improvement was observed, including improvement in general appearance and dyspnea and reduction of the bone pain. The patient no longer required oxygen supplementation. The subcutaneous lesions started to regress too, with complete resolution by the 12th week as well as improvement in all bone lesions (Figs. 1 and 2).

The patient continued on nivolumab until June 2015, and tolerated the treatment well. Unfortunately, she developed bacterial aspiration pneumonia and passed away in June 2015.

## 3 Discussion

Clinical response patterns observed with immunotherapy differ from those seen with cytotoxic agents. Specifically, initial tumor growth followed by subsequent tumor regression (“pseudoprogression”) has been reported in metastatic melanoma. The underlying mechanism for the phenomenon is either continued tumor growth until a sufficient immune response occurs, or a transient immune-cell infiltrate. Immune-related response criteria have been developed in order to address unconventional response patterns observed with immunotherapy. Their use has revealed an additional 10% of favorable responses and survival in ipilimumab-treated melanoma patients.^[4]^ Unconventional “immune-related” responses were also reported in melanoma patients treated with nivolumab, a fully human IgG4 PD-1 immune check-point inhibitor.^[3]^ Nivolumab received Food and Drug Administration (FDA) approval for second-line treatment of squamous NSCLC based on superior survival rate over docetaxel in the CheckMate 017 trial.^[5]^ The rate of pseudoprogression patterns in immunotherapy-treated lung cancer patients is still unclear.

We describe a case of pseudoprogression of subcutaneous metastasis in a patient with lung squamous cell carcinoma treated with nivolumab. Such pseudoprogression may serve to predict subsequent clinical response.
